# Glandular fever and pulmonary artery thrombosis in a paraplegic patient, who had undergone splenectomy for splenic trauma sustained along with spinal cord injury: misdiagnosed initially as urine infection and later as lymphoma when CT scan revealed enlarged lymph nodes: a case report

**DOI:** 10.1186/1757-1626-2-76

**Published:** 2009-01-22

**Authors:** Subramanian Vaidyanathan, Bakul M Soni, Peter L Hughes, David O'Brien, Tun Oo, Wunna Aung

**Affiliations:** 1 Regional Spinal Injuries Centre, District General Hospital, Southport, PR8 6PN, UK; 2 Department of Radiology, District General Hospital, Southport, PR8 6PN, UK; 3 Department of Haematology, District General Hospital, Southport, PR8 6PN, UK

## Abstract

**Background:**

A 36-year-old male sustained fracture of first lumbar vertebra, splenic tear and paraplegia in a motorcycle accident in 2001; splenectomy was performed.

**Case presentation:**

In 2008, he presented with temperature and feeling rough. With a diagnosis of urine infection, he was prescribed ciprofloxacin, followed by trimethoprim, amoxicillin, and gentamicin, as temperature did not subside. White cell count was 21.2 × 10^9^/L; lymphocytes were 13.05 × 10^9^/L (1.00 – 4.00). Therefore, computerised tomography (CT) of chest and abdomen was performed. Thrombus was present in pulmonary arteries bilaterally involving the lobar and segmental branches. Enlarged lymph nodes were seen in axillae, chest, abdomen and inguinal regions. Radiological diagnosis was lymphoma. Cell marker showed an excess of large granular lymphocytes and activated lymphocytes. The Glandular Fever Slide Test was positive. Subsequently, Paul Bunnell test was also positive. Epstein Barr virus serology was consistent with recent Epstein Barr virus infection. Antibiotic was omitted; enoxaparin was prescribed for pulmonary artery thrombosis.

**Conclusion:**

Learning points from this case: (1) Although routine administration of antibiotic to a spinal cord injury patient with pyrexia may be acceptable in outpatient setting, other possibilities such as infection by multi-drug resistant organism, viral infection, venous or, arterial thrombosis should be considered if a patient does not respond promptly to antibacterial therapy. (2) When full blood count showed lymphocytosis (comprising > 50% of white blood cells) with atypical morphology, lymphocyte surface markers, Paul Bunnell test, and Epstein Barr virus serology should be performed. These tests would have led to a diagnosis of infectious mononucleosis, and abdominal imaging studies could have been avoided. (3) Lymphoid hyperplasia is the hallmark of infectious mononucleosis; therefore, we should have suspected glandular fever rather than lymphoma when CT scan revealed enlarged lymph nodes in abdomen, mediastinum, axillae and inguinal regions in this patient, who had lymphocytosis with atypical morphology. (4) A soft tissue mass, situated inferior to left hemidiaphragm in this asplenic patient, was misinterpreted as lymph nodes; review of CT led to the correct diagnosis of splenunculus. (5) Acute infection with Epstein Barr virus may lead to transient induction of anti-phospholipid antibodies, which can cause vascular thrombosis. (6) This case illustrates the value of reviewing test results and discussion with senior doctors, as these measures help to recognize medical errors and improve patient care.

## Background

Despite improvements in medical management of spinal cord injury, re-hospitalisation rates remain high, with an increased incidence in conditions associated with the genitourinary system (urinary tract infections), respiratory complications, and pressure ulcers [1]. Among the reasons for the readmissions of spinal cord injury patients, evaluation and care of urinary tract disorders topped the list of readmission episodes (43.43%) in Northwest Regional Spinal injuries Centre, Southport, UK [2]. Individuals with spinal cord injury have a lifelong increased risk of systemic infection, which may be associated with episodes of life-threatening bacteraemia. A retrospective review of positive blood cultures collected over a 32-month period in chronic spinal cord injury patients treated at the Veterans Affairs Medical Centre Spinal Cord Injury Unit, Memphis, Tennessee, USA revealed 123 episodes of bacteraemia in 63 patients during 83 hospitalizations [3]. The commonest source of bacteraemia was urinary tract infection (n = 39). As spinal cord injury patients are at high risk for developing bacteraemia from urine infections, current practice in Southport spinal unit is to administer antibiotics when a spinal cord injury patient develops fever > 38.5° Celsius. In outpatient clinics, oral antibiotics e.g. trimethoprim, ciprofloxacin or, amoxicillin are prescribed pending results of urine microbiology.

We report a spinal cord injury patient, who attended outpatient clinic with temperature and was prescribed antibiotics with a presumptive diagnosis of urine infection. Subsequently, full blood count revealed lymphocytosis; therefore, computerised tomography of chest and abdomen was performed. Enlarged lymph nodes were seen in axillae, mediastinum, abdomen and inguinal regions; the overall picture was suggestive of lymphoma. However, subsequent blood tests led to a diagnosis of glandular fever, which was a great relief to the patient and his family. We discuss what we learned from this case.

## Case presentation

A 36-year-old male sustained fracture of first lumbar vertebral body with displacement of bony fragment and dislocation at T12/L1 vertebral body, fracture of left ribs (second to twelfth) and left scapula, sub-capsular splenic tear, and paraplegia at Thoracic 12 level in a road traffic accident in October 2001. He underwent laparotomy; multiple lacerations to spleen at the level of the hilum both on gastric and parietal aspects were found. Attempts to salvage the spleen were unsuccessful; therefore, splenectomy was performed. He received pneumococcal and H. influenza vaccinations, and antibiotic prophylaxis. He was ventilated in the post-operative period. Tracheostomy was performed. Later, surgical fixation of dislocated first lumbar vertebra was carried out. He was discharged home in July 2002.

This patient attended outpatient clinic on 07 August 2008 with history of feeling rough and temperature. He was managing neuropathic bladder by intermittent catheterisation. With a presumptive diagnosis of urine infection, this patient was prescribed ciprofloxacin 500 mg, twice daily. Two days later, he developed headache and stomach pain. Therefore, he stopped ciprofloxacin and started taking trimethoprim instead. A spinal unit doctor reviewed this patient over telephone on 13 August 2008. This patient continued to have headache; he had no appetite, he had no energy; he had slight cough; his temperature had been up and down; his tongue was yellow. He was advised to come to spinal unit for urine and blood tests.

This patient attended spinal unit two days later. He was still getting headache; he was feeling slightly unwell. He did not have any symptom pertaining to his chest. He did not have shortness of breath or haemoptysis. A sample of urine was sent for microbiology. X-ray of kidneys and urinary bladder was taken to look for urinary calculi. This patient was advised to take amoxicillin 500 mg, three times a day. The attending doctor discussed with the patient that if microbiology report of urine showed growth of bacteria, and if the organism was resistant to amoxicillin, he would require appropriate antibiotic therapy as indicated by microbiology.

This patient was reviewed over telephone three days later. He continued to feel unwell. Therefore, he was admitted to spinal unit on the same day. Indwelling urethral catheter was inserted as a temporary measure. He was given fluids intravenously. He was prescribed amoxicillin and gentamicin intravenously. Urine microbiology showed no growth. X-ray of kidneys and urinary bladder revealed no radio opaque calculi.

Blood tests of 18 August 2008 showed C-reactive protein of 61.4 mg/L (0.0 – 10.0). Blood urea: 2.5 mmol/L. Creatinine: 88 umol/L. Total bilirubin: 12 umol/L. Alkaline phosphatase: 125 u/L (40–120). Alanine aminotrasferase: 61 u/L (5–37). Gamma glutamyl transferase: 125 u/L (0–50). International normalised ratio: 1.0. APTT ratio: 1.20 (0.84 – 1.16). Calcium: 2.20 mmol/L (2.20 – 2.60). Phosphate: 1.17 mmol/L (0.80 – 1.50). Blood culture showed no growth after 72 hours of incubation. Lactic dehydrogenase: 432 mU/L (0 – 250).

Results of full blood counts are given in Table 1. Marked lymphocytosis with atypical morphology was seen, which was suggestive of reactive process. Lymphocyte surface markers were requested for on 26 August 2008. But this report was received on 02 September 2008.

**Table 1:** Results of full blood counts

Date 2008	18/08	19/08	21/08	22/08	30/08	06/09
Haemoglobin (g/dL)	16.4	16.1	15.8	15.0	15.2	15.8

**White cell count **×10^9^/L (4.0 – 11.0)	21.2	17.7	22.2	26.4	10.0	6.8

Platelets x10^9^/L	366	331	279	322	433	615

Neutrophils x10^9^/L (2.00 – 7.50)	4.96	4.37	5.42	3.87	2.39	1.52

**Lymphocytes **×10^9^/L (1.00 – 4.00).	13.05	10.54	13.03	18.13	6.32	4.15

**Monocytes **×10^9^/L (0.20 – 0.80).	0.83	0.65	0.88	0.84	0.59	0.72

**Eosinophils **×10^9^/L (0.03 – 0.40).	0.03	0.05	0.04	0.02	0.03	0.07

**Basophils **×10^9^/L (0.00 – 0.12).	0.35	0.27	0.39	0.59	0.12	0.10

**Figure 1:**
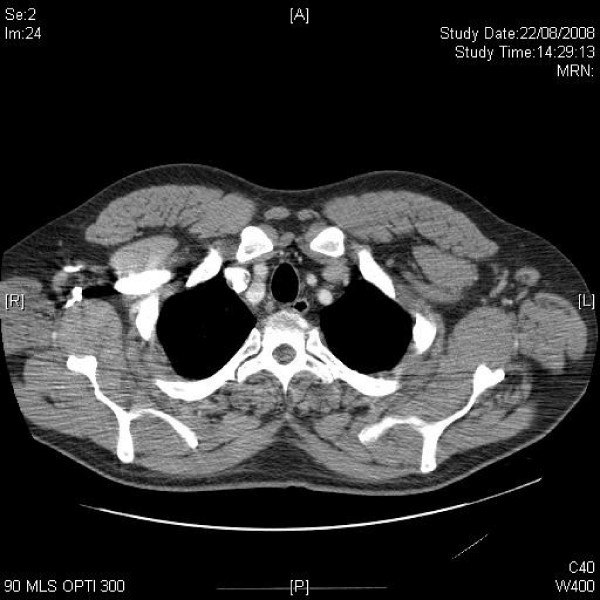
**Axial CT of upper chest demonstrating axillary lymphadenopathy**.

**Figure 2:**
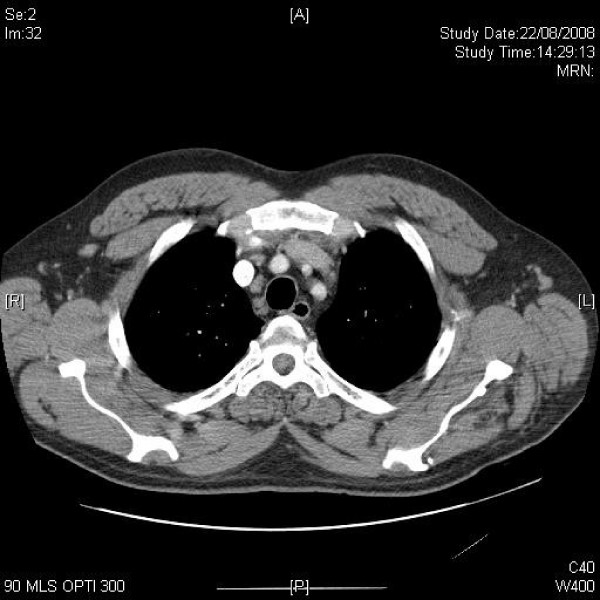
**Axial CT of upper chest shows mildly enlarged axillary and mediastinal lymph nodes**.

**Figure 3:**
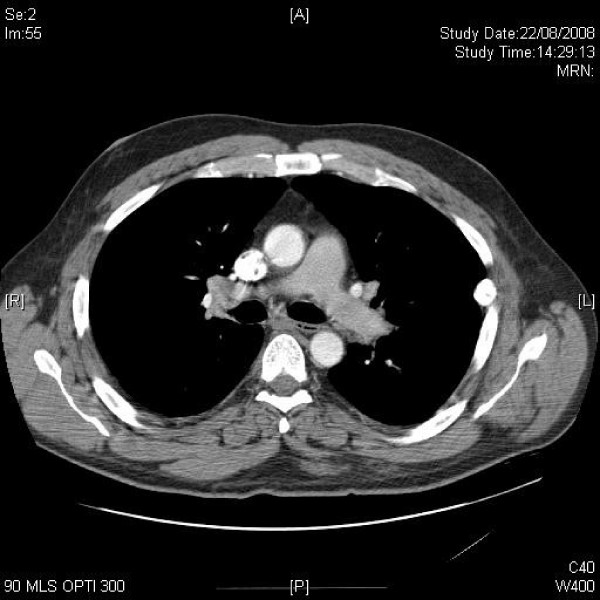
**Axial CT of lung hila shows hilar lymphadenopathy**.

**Figure 4:**
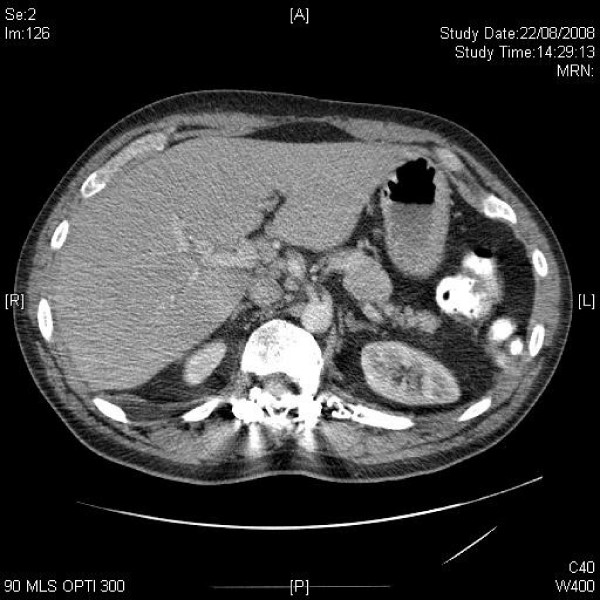
**Axial CT of upper abdomen demonstrates mildly enlarged para-aortic and portal lymph nodes**.

Computerised tomography of chest, abdomen and pelvis was performed on 21 August 2008. CT revealed a one cm left anterior axillary node, subcentimetre nodes in both axilla, multiple subcentimetre mediastinal nodes, the largest one measuring 9 mm in the pre-carina. (Figures 1, 2 and 3) In the abdomen, there was a 3 cm × 2 cm soft tissue mass located inferior to the left hemi-diaphragm, which most likely represented lymph nodal mass. There was a 12 mm node in the aorta-caval region; further subcentimetre nodes were seen in the celiac region around the lesser curvature, paraortic area, and the mesentery. (Figure 4) Lymph nodes were seen in the inguinal region bilaterally measuring 9 mm in the left inguinal area. The overall picture was suggestive of lymphoma.

**Figure 5:**
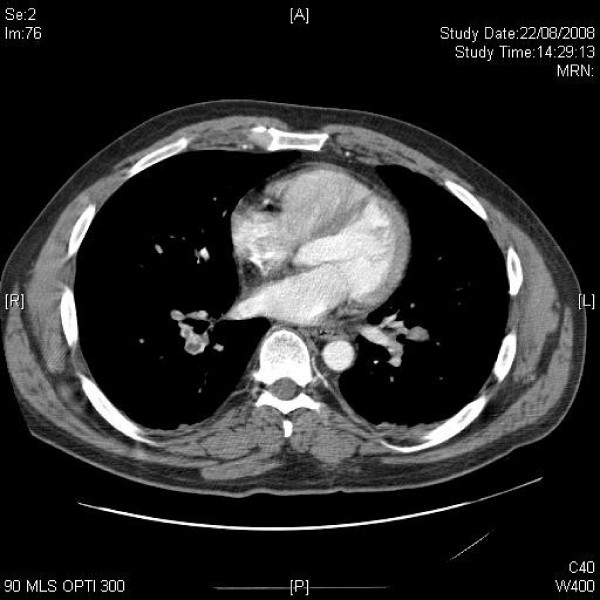
**Axial CT of chest shows embolus in the right lower lobar artery**.

**Figure 6:**
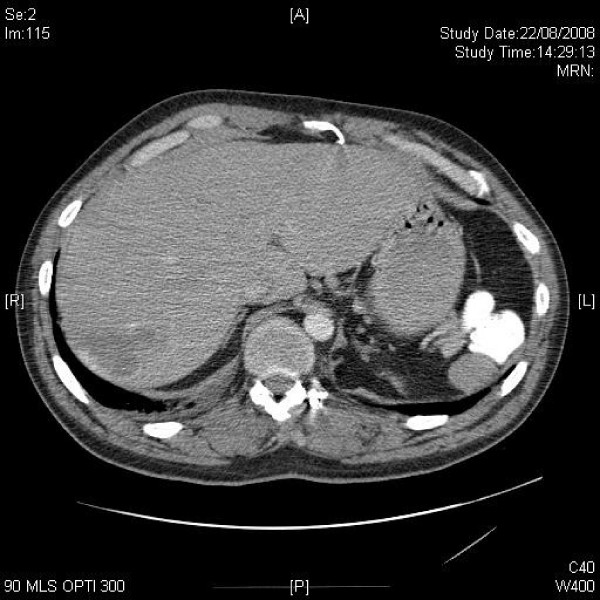
**Axial CT through the liver during arterial phase shows peripheral enhancement of the 6 × 5 cm haemangioma in segment 7**. There is a 3 cm splenunculus on the left side.

**Figure 7:**
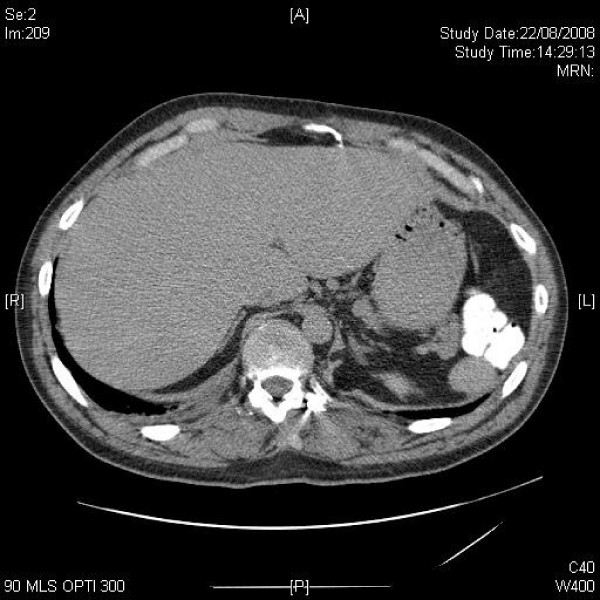
**Axial CT 5 minutes post-contrast demonstrates complete homogenous enhancement of the haemangioma, now being of the same density as normal surrounding liver**. There is a 3 cm splenunculus on the left side.

**Figure 8:**
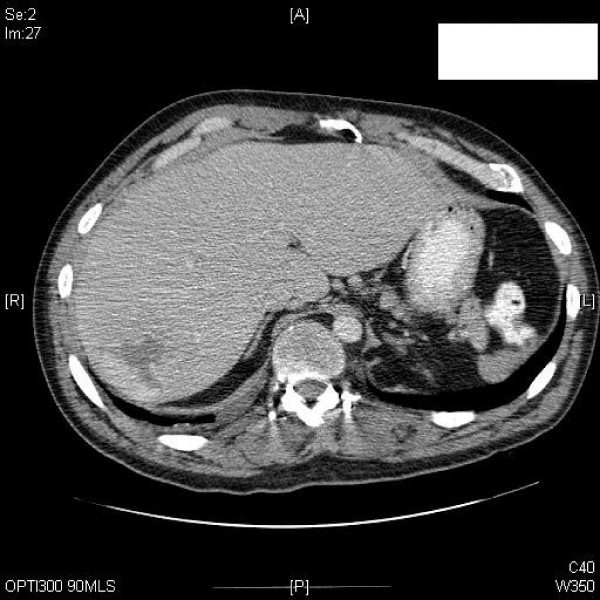
**Axial CT – portal venous phase shows increased enhancement of the haemangioma**.

There was a 4 cm × 2.5 cm irregular area of low attenuation in segment 7 of the liver. In order to assess this lesion, CT with contrast was performed the next day. Computerised tomography with contrast revealed thrombus in the right main lobar branch of pulmonary artery extending into the segmental branch of the lower lobe. (Figure 5) Thrombus was also seen in the segmental branch of right upper lobe, left lower lobe and middle lobe. No lung parenchymal lesion was seen. There was a 6 cm × 5 cm lesion in segment 7 of liver. In the arterial phase, there was peripheral enhancement (Figure 6); the lesion became homogenous with the liver parenchyma in delayed phase. (Figure 7) and increased enhancement was seen in the portal venous phase. (Figure 8) This represented a primary liver lesion but lymphomatous metastases could not be excluded entirely. Therefore, Magnetic Resonance Imaging (MRI) of abdomen was performed on 26 August 2008. MRI revealed a lobulated 5 cm × 4.7 cm lesion in segment 7 of liver, which showed high signal on T2 and fat suppressed sequences. On the post-contrast dynamic sequences, there was peripheral nodular enhancement in the arterial phase with infilling in the portal venous and delayed phases with homogenous enhancement in the late phases. These findings were in keeping with the diagnosis of haemangioma. The nodes noted on previous CT examination in the portal caval (12 mm), para-aortic (subcentimetre), and left hemidiaphragmatic nodes (3 cm) were still present. The radiologist expressed the opinion that lymphoma should be considered.

On 02 September 2008, lymphocyte cell marker showed an excess of large granular lymphocytes and activated lymphocytes. The Glandular Fever Slide Test was positive. There were changes of hyposplenism. On 05 September 2008, Paul Bunnell test was done, which was also positive. Antibiotic was omitted. He was prescribed enoxaparin 1.5 mg/kg subcutaneously for pulmonary artery thrombosis. Epstein Barr VCA IgG was positive; Epstein Barr VCA IgM was positive. These results were consistent with recent Epstein Barr virus infection.

On review of the imaging studies by a senior doctor, hepatic lesion could have been diagnosed as haemangioma by the computerised tomography performed with contrast; MRI scan was perhaps not required. Further, the soft tissue mass located under left hemidiaphragm, which was initially reported as lymph nodes, represented splenunculus. (Figures 6 and 7) The mass received blood supply from splenic artery and venous drainage was by splenic vein.

## Discussion

This patient was prescribed ciprofloxacin, followed by trimethoprim, amoxicillin and gentamicin, as he continued to have temperature. Routine administration of antibiotic to a spinal cord injury patient, who has developed pyrexia, may be acceptable in outpatient setting. But, when a patient does not respond promptly to antibacterial therapy, spinal cord physician should consider other possibilities such as infection by multi-drug resistant organism, viral infection, venous, or arterial thrombosis. In a patient with fever and lymphocytosis, examination of blood film is often a very useful diagnostic aid. Presence of atypical lymphocyte with characteristic features (it is unusually large; it has a scalloped edge where it is indented by erythrocytes; and its cytoplasm is stained dark blue at the edge of the cell) indicates that glandular fever is a likely diagnosis [4] and needs further tests for confirmation. When the initial report of full blood count showed lymphocytosis comprising > 50% of white blood cells, the spinal cord physician should have requested for examination of a blood film. Then, infectious mononucleosis would have been suspected. Abdominal imaging studies (CT chest, abdomen and pelvis, CT with contrast, MRI with contrast) could have been avoided.

In this patient, findings of enlarged lymph nodes in abdomen, mediastinum, axillae and inguinal regions were interpreted as "overall picture suggestive of lymphoma". As this patient had lymphocytosis with atypical lymphocytes, we should have considered the possibility of glandular fever. The diagnosis of lymphoma caused considerable anxiety to the patient and his family. Infectious mononucleosis is usually a self-limited clinical syndrome caused primarily by Epstein-Barr virus infection. The gross changes, which were observed during autopsies of nine patients with infectious mononucleosis, were almost exclusively confined to enlargement of lymphoid tissues [5]. Nasopharyngeal lymphoid hyperplasia was constant, in one instance, suggesting tumour. Lymph nodes were usually but not invariably enlarged. Lymphoid hyperplasia may involve visceral organs as well. Zhang and Molot [6] described an unusual case of acute Epstein-Barr virus infection-associated gastritis with diffuse atypical lymphoid hyperplasia in gastric mucosa.

The classic criteria for diagnosing infectious mononucleosis developed by Hoagland include a lymphocytosis comprising ≥ 50% of the white blood cell differential with atypical lymphocytes accounting for ≥ 10% of the total white blood cell count. Modern haematology analysers are efficient in identifying heterophile-positive patients. Automatic analysers detect quantitative as well as qualitative abnormalities in lymphocytes. Using a combination of all qualitative and quantitative flags, Coulter STKS and Sysmex NE-8000 analysers identified 156 (86.2%) of 181 heterophile-positive cases [7].

This patient had thrombosis involving both pulmonary arteries. Acute Epstein Barr virus infection is associated with transient induction of anti-phospholipid antibodies [8]. In this patient APTT ratio was high; therefore, it is likely that Epstein Barr virus-induced anti-phospholipid antibodies may have played a role in the genesis of pulmonary artery thrombosis.

The 3 cm × 2 cm soft tissue mass, located inferior to left hemidiaphragm was reported initially as lymph node in this patient, who had undergone splenectomy; we failed to suspect splenunculus. Review of CT scan by a senior doctor showed that the mass received its blood supply from splenic artery, and venous drainage was by splenic vein, thus confirming presence of splenic tissue. A diagnosis of splenunculus was reassuring to the patient, as it averted the need for biopsy to exclude neoplastic lesions. Scintigraphy is also useful in detecting splenunculus, Denatured blood cell scintigraphy is considered more sensitive than 99 m Technetium sulphur colloid scintigraphy, and allows imaging of the splenic tissue independent of the liver.

## Conclusion

1. This case is a reminder that sometimes pyrexia in a spinal cord injury patient may be due to causes other than bacterial infection.

2. When full blood count shows lymphocytosis (comprising > 50% of white blood cells) with atypical morphology, lymphocyte surface markers, Paul Bunnell test, and Epstein Barr virus serology should be performed. In this patient, these tests would have led to a diagnosis of glandular fever. Then abdominal imaging studies could have been avoided.

3. Lymphoid hyperplasia is the hallmark of infectious mononucleosis; therefore, presence of enlarged lymph nodes in abdomen, mediastinum, axillae and inguinal regions, as seen in CT scan, should have prompted a diagnosis of glandular fever in this patient, who had lymphocytosis with atypical morphology.

4. CT of abdomen revealed a soft tissue mass inferior to left hemidiaphragm in this asplenic patient, which was misinterpreted as lymph nodes, whereas the mass represented splenunculus; review of CT revealed that the blood supply was from splenic artery and venous drainage was by splenic vein.

5. Acute infection with Epstein Barr virus may lead to transient induction of anti-phospholipid antibodies, which can cause vascular thrombosis.

6. This case illustrates the value of reviewing test results and discussion with senior doctors, as these measures help to recognize medical errors and improve patient care.

## Competing interests

The authors declare that they have no competing interests.

## Authors' contributions

SV developed the concept and wrote the draft. PH reviewed medical images. DO reviewed haematology results. All authors contributed to patient care.

## Consent

Written informed consent was obtained from the patient for publication of this case report and accompanying images. A copy of the written consent is available for review by the Editor-in-Chief of this journal.
